# AutInsight: A Pilot Randomised Controlled Trial (RCT) of a Consumer-Informed Parent Support Program for Parents of Autistic Children

**DOI:** 10.1007/s10803-025-06764-5

**Published:** 2025-02-27

**Authors:** Jia Ying Sarah Lee, Koa Whittingham, Amy E. Mitchell

**Affiliations:** 1 Queensland Cerebral Palsy and Rehabilitation Research Centre, UQ Child Health Research Centre, The University of Queensland, Brisbane, QLD 4101 Australia; 2 School of Nursing, Midwifery and Social Work, The University of Queensland, Brisbane, Australia; 3 Parenting and Family Support Centre, The University of Queensland, Brisbane, Australia; 4 Griffith Centre for Mental Health, Griffith University, Brisbane, Australia

**Keywords:** Autism, Parenting, Support, Children, Acceptance

## Abstract

AutInsight is an innovative, consumer-informed parent support program for parents of autistic children grounded in perspectives from qualitative research with autistic adults. The current study outlines the development and evaluation of AutInsight through a pilot randomised controlled trial. Parents (*N* = 41) of autistic children (10 years and younger) were randomly allocated to AutInsight (*n* = 20) or care-as-usual (*n* = 21) and completed online questionnaires across three timepoints (baseline, post-program and 3-month follow-up). Measures include parental sensitivity, parental acceptance and understanding, psychological flexibility, mindful parenting, parental mental health, overall family experience, quality of life, and child behaviours. Intent-to-treat analyses indicated greater rates of improvement for parents allocated to the AutInsight program, with small- to medium-sized effects for parent-reported parental sensitivity (EA-SR mutual attunement *d* = 0.84, EA-SR child involvement *d* = 0.50), as well as overall autism family experience (AFEQ_total_ *d* = 0.38, AFEQ_family life_ *d* = 0.29). Greater rates of improvement were also found in parent-reported child conduct problems (SDQ *d* = 0.62) and prosocial behaviours (SDQ *d* = 0.48). No significant results were found in observed parental sensitivity or any other measure. Results suggest preliminary evidence of effectiveness, feasibility and acceptability of the AutInsight program. A larger, fully powered trial is warranted. This trial was prospectively registered with the Australian and New Zealand Clinical Trials Registry (Trial ID: ACTRN12623000806662).

**Supplementary Information:**

The online version contains supplementary material available at 10.1007/s10803-025-06764-5.

## Introduction

Autism (termed autism spectrum disorder in the DSM-5 TR) is a lifelong neurodevelopmental disability characterized by differences in social emotional communication as well as restrictive and repetitive behavioural patterns (Baron-Cohen, 2008). Autistic children experience higher rates of externalising and internalising behaviours (Micai et al., 2023), and continue to experience higher rates of anxiety, depression, and suicidal ideation as adults (Croen et al., 2015). Parenting an autistic child can be challenging, different from what the parent may have expected, and has potentially lifelong implications for both the parent and child (Gau et al., 2010; Ventola et al., 2017). Parents of autistic children experience more stress and mental health challenges than parents of children with other disabilities (Blacher & McIntyre, 2006). Additionally, many parents of autistic children are themselves autistic which may pose additional challenges. Therefore, they require support, both in managing their own well-being and in managing the daily challenges of parenting an autistic child.

Participatory research, involving community stakeholders, is important as it ensures that the needs of the community are met. However, no parent program has, to our knowledge, been created based on consultation with autistic people on what was important within their own parent–child relationships and what *they* wanted from their parents as autistic children. The program tested in this study was developed grounded in a qualitative study by Lee et al. (2023), where autistic adults were asked about their experiences of being parented in childhood and the advice they would provide to present day parents within the context of their lived experience. Autistic adults reported that they wanted unconditional acceptance and love, within a holistic understanding of their specific autistic profile. They wanted their parents to know that their “goal is happiness, not neurotypical success”, and that parents needed to collaboratively figure out what that “happiness” might look like for their specific child and support them in those directions without suppressing their child’s autistic self.

Feeling accepted and loved by one’s parents is a key part of the parent–child relationship. Parental acceptance is important to children’s attachment security (Ildiz & Ayhan, 2022) as well as a child’s emotional and social development (Rohner & Britner, 2002; Rohner et al., 2005). Research within the mental health literature suggests that perceived acceptance and belonging (or the lack thereof) from one’s close networks has significant impacts on autistic mental health (Cage et al., 2018; Milton & Sims, 2016). Acceptance from others predicts less depression and stress in autistic adults, whereas the lack thereof contributes to greater “camouflaging”, defined as strategies to hide or disguise autistic differences to better fit in (Cook et al., 2021), and is associated with greater symptoms of depression (Cage et al., 2018). While parental acceptance of their child’s autism diagnosis, otherwise known as “diagnosis resolution”, has received some attention in the literature (Naicker et al., 2022), there is a dearth of research on parental acceptance of the *whole* autistic child. Consequently, no research has built a program grounded in what autistic adults think is important in parenting.

Acceptance and commitment therapy (ACT) and attachment theory are two theoretical frameworks that can complement each other to support the development of a parent support program grounded in supporting parents to develop acceptance and understanding of their autistic child—what autistic adults said *they* wanted. Autistic adults said that “understanding (their) specific autistic child is crucial to meeting their needs” (Lee et al., 2023). ACT can support parents in developing psychological flexibility and thus their ability to have a flexible and broader understanding of their own and their child’s internal experiences and behaviour through a non-judgmental, curious and accepting perspective (Whittingham, 2014). ACT targets psychological flexibility by targeting components of psychological flexibility, including mindfulness, experiential acceptance and values (Hsu et al., 2023; Macri & Rogge, 2024). Developing a parent’s ability to be present with their child through mindfulness, along with fostering acceptance of said present moment, including the internal experiences (e.g., thoughts and feelings) it brings, enables the parent to truly connect with and be insightful in the moment. It is from this place of mindful, context-dependent understanding of their child’s internal and external behaviour that parents can ground their responses in, and attune to, their child’s needs.

Autistic adults also underscored the importance of feeling emotionally safe in their relationship with their parents—to be accepted and seen for who they are (Lee et al., 2023). Attachment theory provides a platform where we can understand autistic children’s attachment needs of safety and security as well as how parents’ own attachment history can influence parent–child relationships, impacting on parents’ capacity and ability to be sensitive and accepting of their child. Developing a parent’s understanding and awareness of the relevance of attachment theory—both in relation to themselves, their relationship with their child, and their own attachment figures—reveals the reciprocal nature of these relationships. This awareness ultimately impacts their ability to connect and relate authentically with the child in front of them. There are parent–focused ACT-based programs for parents of children with disabilities (Whittingham et al., 2020), and attachment-based programs for parents of autistic children (Kubo et al., 2021; Salman, 2016), however, no research has created a parent support program grounded in ACT and attachment theory where the primary focus is on the quality of the parent–child relationship, as understood by autistic adults. Further, no study has grounded their parenting program on what autistic adults said they wanted.

Hence, the aim of this study was to test the efficacy, feasibility and acceptability of a consumer-informed parent support program through a pilot randomised controlled trial (RCT) of *AutInsight*: a program developed based on insights and advice from autistic adults who wanted acceptance and understanding from their own parents as children (Lee et al., 2022, 2023) as well as involvement from an autistic reference group. Grounded in attachment theory and ACT, *AutInsight* seeks to help parents to (a) develop greater insight and understanding of autism from the inside out, drawing on the experiences and perspectives of autistic adults; (b) strengthen their relationship with their child; and (c) develop practical strategies for parents’ self-care. Compared to care as usual (CAU), participation in AutInsight—a 5-week online group program designed to improve parental acceptance, understanding and sensitivity—was hypothesised to:

### Primary Outcomes:

- [H1a] increase observed and self-reported parental sensitivity (emotional availability) as assessed by the Emotional Availability (EA) Scales (Biringen, 2022) and Emotional Availability Self-Report (EA-SR) (Biringen et al., 2002)
- [H1b] increase observed and parent-reported child responsiveness and child involvement toward their caregiver as assessed by the EA Scales (child responsiveness and child involvement scales) and EA-SR (Biringen, 2022)

### Secondary Outcomes:

- [H2] increase acceptance and understanding of parents towards their autistic child as assessed by the Parental Acceptance and Understanding of Autistic Children Scale (PAUACS) (Lee et al., 2024)
- [H3] increase psychological flexibility of parents as assessed by the Comprehensive assessment of Acceptance and Commitment Therapy processes (CompACT) (Francis et al., 2016)
- [H4] increase mindful parenting as assessed by the Bangor Parenting Mindful Parenting Scale (BMPS) (Griffith & Hastings, 2022)
- [H5] improve parental psychological adjustment as assessed by the Depression, Anxiety, Stress Scales (DASS-21) (Lovibond & Lovibond, 1995)
- [H6] improve child adaptive functioning, parental mental health and parental wellbeing as assessed by the Autism Family Experience Questionnaire (AFEQ) (Leadbitter et al., 2018)
- [H7] increase parental quality of life as assessed by the Quality of Life in Autism Questionnaire (QoLA) (Eapen et al., 2014)
- [H8] reduce child behavioural difficulties as assessed by the Strengths and Difficulties Questionnaire (SDQ) (Goodman, 1997), and
- [H9] increase child behavioural flexibility as assessed by the Behaviour Flexibility Rating Scale – Revised (BFRS-R) (Peters-Scheffer et al., 2008).

In addition, we assessed whether the AutInsight program was feasible to implement and acceptable to parents via recruitment, retention, and adherence rates, as well as qualitative parent feedback.

## Method

### Study Design and Procedures

**Fig. 1:**
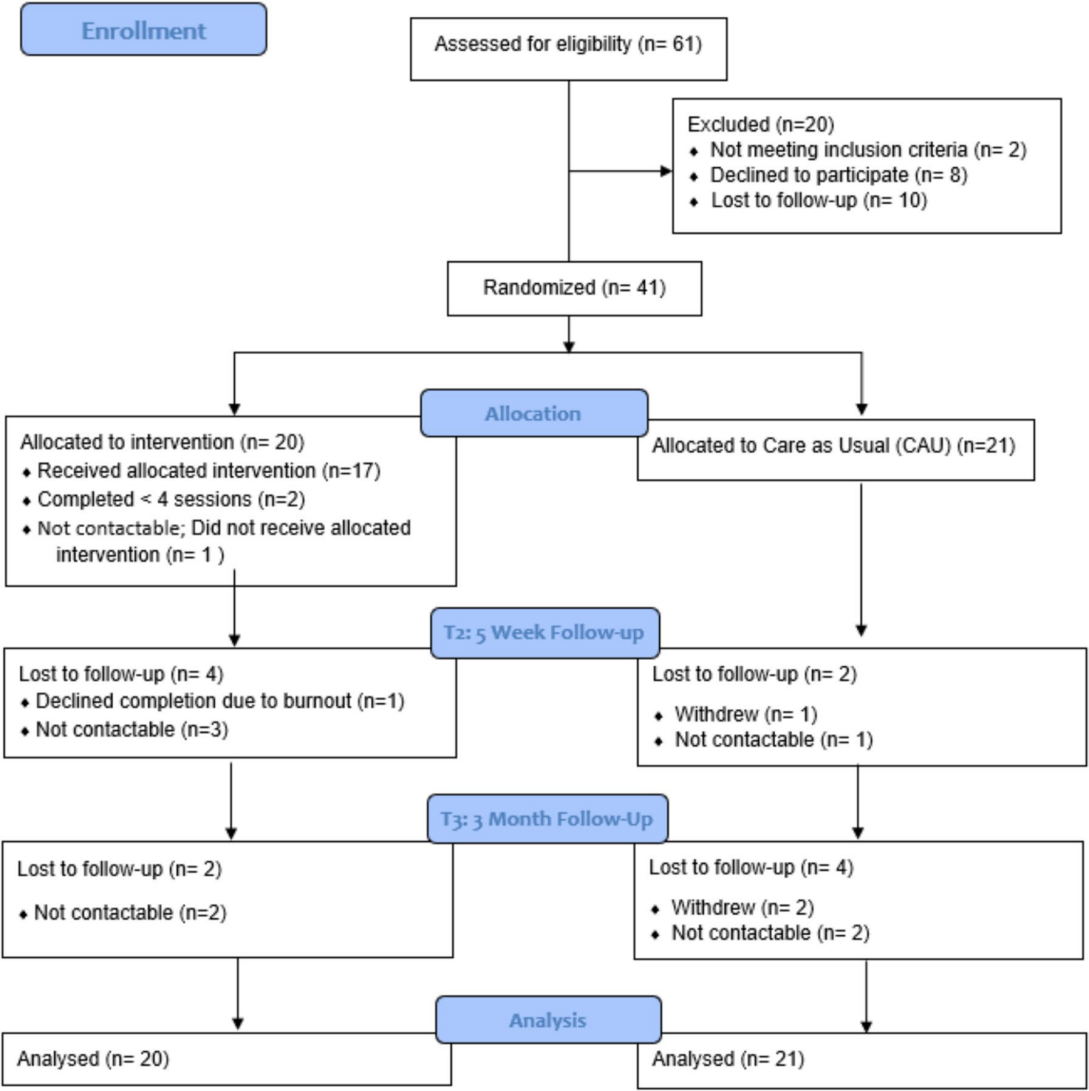
CONSORT flow diagram

A 2 (AutInsight vs. Care as Usual [CAU]) × 3 (time: baseline [T1], post-intervention [T2], 3-month follow-up [T3]) design was used. This RCT tested the relative impact of AutInsight against CAU on parent and child outcome measures. Families were assessed at baseline on all variables, and then randomised to one of the two conditions using computer-generated randomly selected block sizes and random group allocation within each block. The computer database (RedCap) generated random allocation sequences. Group allocation was therefore independent of the researcher and allocated to families in the order in which baseline assessment was completed. Refer to Fig. 1 for the study design.

Participants were parents of autistic children (aged 10 years and younger) who resided in Australia. Families were asked to assign one parent as the “primary participant” (usually the main caregiver, or the parent who spends most time with the child) who completed all assessments and attended program sessions. The other parent was also encouraged to attend sessions if they wanted to. Where families had multiple autistic children, parents were asked to select the child that they were most concerned about to be the focus child for the purposes of the study. Participants were recruited through autism support organisations, mailouts to Autism-related organisations, social media (e.g., Facebook, Instagram, Reddit), and word of mouth. This trial was prospectively registered with the Australian and New Zealand Clinical Trials Registry (Trial ID: ACTRN12623000806662). Ethical approval was obtained from the Human Research Ethics Committee of The University of Queensland (2023/HE000414) prior to recruitment. Recruitment took place between September 2023 and February 2024. Follow-up data collection was completed by August 2024.

Eligibility was assessed according to the following inclusion criteria: (a) parent of child aged 10 years or younger, who is autistic (i.e., diagnosed with autism spectrum disorder, [ASD], Asperger syndrome or autism); (b) resides in Australia; (c) able to commit to a fixed 2-h timeslot weekly for 5 weeks; (d) access to a device with the ability to access reliable internet; and (e) willing to be randomised to either the AutInsight program or CAU group. Participants were excluded from the study if their child had not received a formal diagnosis at time of enrolment or they were looking for parent programs that taught specific behavioural strategies. Participants were provided a brief description of the study via mail outs and study advertisements and directed to the study website for more information. Via the study website, interested parents contacted the first author should they wish to participate. A phone screening process was conducted to confirm eligibility, provide further information about the study, and answer any queries or concerns parents had. Participants who met eligibility criteria were provided with a copy of the study information sheet and consent forms to complete online. The information sheet outlined in detail the purpose and duration of the study, information about the program, randomisation requirements, participant confidentiality and their rights and ability to withdraw at any time. Participants who returned the signed consent form were then invited to complete the baseline assessment, comprising a set of questionnaires (completed online via Redcap) and a video recorded parent–child interaction observation assessment (via Zoom).

Five weekly, 2-h, online program sessions were provided to the AutInsight participants. Sessions were conducted over consecutive weeks unless there was a public holiday in which case the session was rescheduled to the following week. Group sizes ranged between two to six parents. Two families were not able to attend any of the scheduled groups, even though they previously mentioned they could make the times and were offered 1-to-1 sessions instead. Attendance was noted and all sessions were recorded for protocol fidelity and clinical supervision purposes. Six weeks post T1 assessments, participants from both groups completed T2 assessments. Ten weeks post T2 assessments, participants from the AutInsight and CAU groups completed their 3-month follow-up assessments. Participants in the CAU group continued their care as usual while waiting for the program and were then offered access to the program after completing follow-up assessments.

### Measures

Assessments were conducted at three timepoints, which takes about 30–40 min to complete each. All parents provided demographic information at T1. Parents allocated to the AutInsight condition completed a client satisfaction questionnaire at T2. All other measures were to be completed by all families at each timepoint (T1, T2, T3).

### Sociodemographic Characteristics

Parents’ sociodemographic information, including age, biological sex, transgender identity, relationship status, educational and employment status, highest educational qualification, and income was collected. Children’s ages and their diagnostic information were also collected. Parents were also asked about whether they themselves were autistic.

### Measures—Primary Outcomes

**Emotional Availability Scales (EAS) [H1].** The EA Scales (4 ed.)(Biringen, 2022) is an observational assessment of the dyadic emotional availability of an adult towards the child, and of the child towards the adult. There is a total of four adult scales (sensitivity, non-intrusiveness, structuring, and non-hostility) as well as two child scales (responsiveness, involvement of adult). Greater overall total scores represent greater emotional availability towards the child. Parent–child video observations were conducted via Zoom for 20–25 min each. Parents were sent an information sheet on instructions on how to set up the device prior to the recording to ensure that both parent and child were in view. The EAS has been used with autistic children and their caregivers (Bentenuto et al., 2020; Sher-Censor et al., 2024). The first author (JYSL) scheduled and conducted all recordings. One independent researcher scored all videos, and another researcher (KW) coded 20% of the videos for inter-rater reliability purposes. Inter-rater reliabilities were assessed via assessing the ICC of the scores between both raters and they were all > 0.70.

**Emotional Availability—Self-Report (EA-SR) [H1].** The EA-SR (Biringen et al., 2002) is a 36-item self-report scale assessing parental emotional availability towards their child and the parent–child relationship. Participants respond to a series of statements on a five-point Likert scale: 1(Not agree at all), 2(Rather not agree), 3(Neutral), 4(Rather agree), 5(Totally agree). The EA-SR comprises of five scales: Mutual Attunement, Child Involvement, Affect Quality, Intrusiveness, and Hostility. Greater scores indicate greater mutual attunement, child involvement, affect quality, intrusiveness, and hostility, respectively. Greater total scores represent greater emotional availability towards the child and the parent–child relationship. Internal consistency was between 0.32 and 0.94 in the current sample: Mutual Attunement (*α* = 0.77), Child Involvement (*α* = 0.88), Affect Quality (*α* = 0.32), Intrusiveness (*α* = 0.64), and Hostility (*α* = 0.94). Given the low (< 0.60) internal consistency of Affect Quality, a decision was made to exclude this subscale in the analyses.

### Measures—Secondary Outcomes

**Parental Acceptance and Understanding of Autistic Children Scale (PAUACS) [H2].** The PAUACS (Lee et al., 2024) is a 30-item self-report scale assessing parental acceptance and understanding of their autistic child. Parents respond to items on a seven-point Likert scale: 1(Strongly Disagree), 2(Disagree), 3(Somewhat Disagree), 4(Neutral), 5(Somewhat Agree), 6(Agree), 7(Strongly Agree). There are a total of four scales: Understanding (*α* = 0.74), Innate (*α* = 0.75), Acceptance (*α* = 0.77), and Expectations (*α* = 0.47). Higher scores on the PAUACS represent greater parental acceptance and understanding. Given the low (< 0.60) internal consistency of the Expectations subscale, a decision was made to exclude this subscale in the analyses.

**Comprehensive assessment of Acceptance and Commitment Therapy processes (CompACT) [H3].** The CompACT (Francis et al., 2016) is a 23-item self-report questionnaire assessing psychological flexibility. There are a total of three scales: Openness to Experience (OE), Behavioural Awareness (BA) and Valued Action (VA). Participants rate each item on a seven-point Likert scale: 1(Strongly Disagree), 2(Disagree), 3(Somewhat Disagree), 4(Neutral), 5(Somewhat Agree), 6(Agree), 7(Strongly Agree). Higher scores on the CompACT indicate greater psychological inflexibility. The scales demonstrated high levels of internal consistency across scales within this sample (OE *α* = 0.85; BA *α* = 0.83; VA *α* = 0.82).

**Bangor Mindful Parenting Scale (BMPS) [H4].** The BMPS (Griffith & Hastings, 2022) is a 15-item scale assessing mindful parenting in parents of children with intellectual disability and/or autism. Participants rate each item on a four-point Likert scale: 0(Never true), 1(Sometimes True), 2(Often True), 3(Always True). Higher scores indicate greater mindful parenting. The scale demonstrated good reliability within this sample (*α* = 0.85).

**Depression Anxiety and Stress Scales −21 (DASS-21) [H5].** The DASS-21 is the shortened version of the DASS (Lovibond & Lovibond, 1995) comprising 21 items to assess symptoms of depression, anxiety and stress amongst adults. Participants are asked to think about their experiences in the past seven days and to judge how much each statement applied to them. There are four response options: 0(Did not apply to me at all–never), 1(Applied to me to some degree, or some of the time–sometimes), 2(Applied to me to a considerable degree, or a good part of time–often) or 3(Applied to me very much, or most of the time–almost always). The DASS has been found to have good reliability (Cronbach’s α for Depression = 0.95, Anxiety = 0.91, and Stress = 0.92) in this sample, and validity in clinical and non-clinical samples (Antony et al., 1998). DASS-21 scores were doubled to obtain DASS-42 scores for comparison with norms of the DASS-42.

**Autism Family Experience Questionnaire (AFEQ) [H6].** The AFEQ (Leadbitter et al., 2018) is a 48-item questionnaire developed to assess autistic families’ experience and quality of life. It was originally developed for the purposes of assessing the impact of interventions. There is a total of four sub-scales: (i) experience of being a parent of a child with autism, (ii) family life, (iii) child development and (iv) child symptoms. Participants rate each item on a five-point scale if relevant or “N/A” if not applicable to their family: 1(Always), 2(Often), 3(Sometimes), 4(Rarely), 5(Never). The AFEQ demonstrated excellent reliability across scales in the current sample: parent’s experience (*α* = 0.89), family life (*α* = 0.81), child development (*α* = 0.82) and child symptoms (*α* = 0.69). Overall, the total scale also had excellent reliability (*α* = 0.92) (Leadbitter et al., 2018).

**Quality of Life in Autism Questionnaire (QoLA) [H7].** The QoLA (Eapen et al., 2014) is a 48-item questionnaire developed to assess autism-specific quality of life for parents and caregivers of autistic children. There are two subscales: (i) perception of quality of life (Part A), where participants rate each item on a five-point Likert scale: 1(Not very much) to 5(Very much), and (ii) parent report of how problematic their child’s autism-specific difficulties are for them (Part B), where participants rate each item on a five-point Likert scale: 5(Not much of a problem for me) to 1(Very much of a problem for me). This scale has strong concurrent validity with other quality of life measures (e.g. WHOQOL-BREF; Eapen et al., 2014) and excellent reliability for both Part A (α = 0.96) and Part B (α = 0.92) in this sample.

### Child Outcome Measures

**Strengths and Difficulties Questionnaire (SDQ) [H8].** The SDQ (Goodman, 1997) is a 25-item questionnaire developed to assess behavioural strengths and difficulties in children between the ages of 2–17 years. There is a total of five scales: emotional symptoms (*α* = 0.72), conduct problems (*α* = 0.60), hyperactivity/inattention (*α* = 0.74), peer relationship problems (*α* = 0.59) and prosocial behaviour (*α* = 0.81). Participants rate each statement on a 3-point scale: 0(Not true), 1(Somewhat true) and 2(Certainly true). The SDQ has demonstrated good psychometric properties in neurotypical (*α* = 0.73) (Goodman, 2001) and autistic (α = 0.52–0.81) people (Findon et al., 2016). It also demonstrated good internal consistency in this sample as noted above.

**Behaviour Flexibility Rating Scale—Revised (BFRS-R) [H9].** The BFRS-R (Peters-Scheffer et al., 2008) is a 16-item rating scale for measuring behavioural flexibility in autistic children and related developmental disabilities in specific situations. Participants are asked to rate the severity of each potentially problematic situation (e.g. “Materials break, causing a premature end to an activity”) on a three-point Likert scale, ranging from 0(Not a problem at all) to 2(The situation causes severe problems). A higher score indicates greater difficulties with behavioural flexibility. There are three subscales: (i) Flexibility towards objects (*α* = 0.83), (ii) Flexibility towards the environment (*α* = 0.80), and iii) Flexibility towards persons (*α* = 0.61). This scale demonstrated excellent internal consistency for the total scale (α = 0.88) in this sample.

### Treatment Acceptability, Fidelity, and Feasibility

Treatment acceptability was determined via a feedback survey completed by AutInsight parents at the end of the program (T2) to gather participants’ perceptions of relevance and usefulness. Items included questions such as “How likely are you to recommend this program to another parent of an autistic child?”. Parents rated items on a 10-point Likert scale ranging from ‘Not likely at all’ to ‘Very likely’. Open-ended questions such as “Was there anything you would change about the program?” were also included. Acceptability was also determined by the proportion of participants who were likely or very likely to recommend this program to another parent and how neurodiversity-affirming they found the program. Parents were also asked to report on whether they felt their insightfulness, acceptance and self-care had increased, stayed the same or had decreased since starting the program. They were also asked to explain what they thought brought about that change and to provide some examples. For example, “Do you feel like you have greater insight about the child you have identified?”.

Treatment fidelity was evaluated by having an independent researcher review video recordings of a randomly selected sample of sessions and rate whether each learning objective of each session was met, partially met, or not met. These ratings were compared to checklists which were completed by the facilitator after each session to record completion of session content and achievement of session objectives. The facilitator (JYSL) completed fidelity ratings, while another researcher (KW) completed fidelity ratings for 25% of the sessions, and percent agreement was calculated.

Program feasibility was determined through the number of families who assessed the study website, contacted the first author for more information, were assessed for eligibility and were eventually randomised and attended sessions. Attendance records were also examined to assess for acceptability of the program.

## Development and Implementation of AutInsight

### AutInsight Protocol

The AutInsight program was developed grounded in qualitative research with autistic adults, with the aim of supporting parents of autistic children to develop insight and understanding into their specific autistic child so that they could provide what autistic adults said they wanted from their parents: acceptance and understanding of their child’s whole autistic self, and to ground their parenting based on this (see Supplementary Materials). The program manual was developed by the first author with input from the second author and was informed by acceptance and commitment therapy (ACT) (Hayes, 2004; Hayes et al., 2011), attachment theory (Ainsworth, 1978; Bowlby, 1982), and systemic, humanistic, and person-centred therapy principles (Tudor & Worrall, 2006). The program supports parents in developing insightfulness towards their specific autistic child, facilitated through ACT exercises grounded in a compassionate understanding of the unique parent–child attachment system.

In addition, the findings from the qualitative study were shared as a psychoeducation exercise. Themes from Lee et al.’s (2023) study were translated into program components. The program is situated within the insights of the autistic community—parenting grounded in an understanding of the child and acceptance. The program involved psychoeducation, discussions, reflective and experiential exercises on various topics including autism, acceptance, and how the different skills of mindfulness, acceptance and flexibility can support parents in grounding their parenting in an understanding of their specific child. For example, in Session 4: *Focus on the Relationship and What Works*, parents are encouraged to build on the mindfulness and acceptance skills introduced in earlier sessions. They are invited to harness awareness and curiosity in the present moment with their child. For example, in a particular exercise designed for this program, parents were presented with a novel image and guided through a mindfulness exercise. The image used in this pilot study was a microscopic composite of single-cell freshwater protozoa. A novel picture was selected to facilitate mindfulness, as unfamiliar stimuli are less likely to evoke pre-existing assumptions or interpretations. Additionally, the object was assigned a nonsense label, “Tesnus” for similar reasons. Following the mindfulness exercise, parents participated in a guided discussion and debrief, reflecting on their ability to engage in a “beginner’s mind” perspective. Parents were then asked to bring up a personal photo of their child and were guided through a similar mindfulness process. The script for this exercise can be found below:

*“Mindfulness does not need to be extravagant or take up a lot of time. It is a process or a “skill” rather than something to be achieved. This will be illustrated through some exercises today, and how you can apply mindfulness to your parenting too.*

*In a moment, I am going to show you a picture. I call it a Tesnus. For many of you, this will be something you’re seeing for the first time. However, if you have seen it before I’d like you to imagine that it is something you’re seeing for the first time. I’ll be guiding you through the exercise with some prompts, I’d like you to keep your responses in your mind and we’ll debrief after the exercise.*

*“Let’s start by getting into a comfortable position and taking a couple of slow deep breaths. In through the nose and out through the mouth… Keeping your eyes open.”*

*[Picture shown]*

*“This is a Tesnus. What do you see in this picture?*

*What colours do you see? [Pause] Notice that there are many colours – Blue, white, yellow, black, orange, purple. There are also almost different shades to each of these colours. Some darker, some lighter.*

- *What else do you notice about the colours?*
- *What sorts of textures do you see?*
- *Are there smooth edges? Rough edges?*
- *How would you describe the textures if they’re not smooth or rough?*
- *How about shapes? What sorts of shapes do you see in this picture?”*

*[Debrief]*

- *How was the process for you?*
- *That was an extended exercise of a mindful moment—but in the same way can you see that it can even be a couple of seconds? Just noticing the things around you for what they are.*
- *What other situations could you apply this to?*
- *What if you noticed everyday things around you, with this beginner’s eye?*
- *What if you noticed your child with this beginner’s eye?*
- *Was there something you noticed in the picture or perhaps yourself, that surprised you?*

*“You might be wondering, how is being mindful with a Tesnus relevant to parenting? Well before I answer this question, I’d like for us to do another exercise.*

*I’ll like all of you to whip out your phones. Go to your photos, and I’d like you to find the last photo you have of your child. Can you notice this moment that you have frozen in time with this photo with a beginner’s mind?*

*Refrain from interpreting beyond the picture, even though you know the context of this photo. I’d like you to imagine it is the first time you’re seeing this photo of your child. What do you see? What colours do you see? What textures do you see? What facial expressions?*

*Notice what happens in your body as you’re looking at this photo. Notice where this feeling comes up in your body. What does this feeling or sensation in your body tell you?”*

*[Debrief]*

- *What was the process like for you this time?*
- *Did you notice something different that you did not notice before?*
- *Was there something that surprised you?*

Presentation slides used in delivery of the program were designed in Canva, with consideration given to colours and designs that were less visually stimulating. Green and brown are preferred by autistic people, while yellow is considered sensory overloading (Grandgeorge & Masataka, 2016). Detailed program manuals were developed, and fortnightly clinical supervision was provided by KW. The first author (JYSL; clinical psychologist), who is experienced in working with autistic people and their families, facilitated all groups with clinical supervision from the second and third authors.

### Statistical Analysis

An estimated 38 participants (19 participants per arm [AutInsight vs CAU]) were required for 80% power and 5% two-tailed significance and a medium effect size (Cohen’s *d* = 0.5) for the primary outcomes, based on recommendations for powering pilot studies from Whitehead et al. (2016) and allowing for up to 20% attrition. A total of 14.9% of data were missing completely at random as assessed via Little’s (1988) MCAR test, and the full information maximum likelihood approach allowed inclusion of all cases in analyses. SPSS v.27 was used for all statistical analyses.

Longitudinal intention-to-treat (ITT) analyses were performed in SPSS v27 using mixed-model repeated-measures (MMRM) hierarchical linear regression models to compare change over time between groups on primary and secondary outcomes across all 3 assessment timepoints. Time was the main predictor and group membership was the main moderator. After assessing for a main effect of time (T1/T2/T3) (fixed and random effects; Model 1), fixed effects of group and group-by-time interactions were added (Model 2) with statistically significant group-by-time interaction terms indicating intervention effects. Follow-up contrasts using fixed and random effects of time as predictors were run separately for AutInsight (Model 3) and CAU (Model 4) groups to interpret the direction of intervention effects, and t-tests compared rate of change (regression parameter estimate for time) between groups. Cohen’s *d* indicated effect size (Cohen, 1988). Where data variability was insufficient to support inclusion of random effect of time, the random effect was excluded from all four models. Analysis was conducted for all participants by originally assigned group.

## Results

### Participants

Overall, 61 parents of autistic children expressed interest and consented to be contacted for screening. Of the 61 parents, 41 (67.2%) parents met inclusion criteria, completed baseline assessments, and were randomised into the trial. A total of 20 parents were excluded as they did not meet inclusion criteria (*n* = 2), were not contactable (*n* = 10) or declined to participate due to time commitment (*n* = 7), or the program was not what the parent wanted (*n* = 1) (see Fig. 1). The overall sample therefore comprised of 41 parents (AutInsight *n* = 20 [5 mother-father dyads, 15 individual parents]; CAU *n* = 21 [0 mother-father dyads, 21 individual parents]).

Participant characteristics are presented in Table 1. Children were aged 1–10 years (mean age = 6.41 years, *SD* = 2.47), with the majority assigned male at birth (*n* = 30, 75.0%). Most primary caregivers were mothers (*n* = 36, 87.8%), who were born in Australia (*n* = 31, 75.6%). No participants identified as gender diverse. Parents were aged 25–54 years (mean age = 39.71 years, *SD* = 5.63). Most identified English as their first language (*n* = 38, 92.7%), while the minority reported Mandarin (*n* = 1), Arabic (*n* = 1) or Spanish (*n* = 1) as their first language. Most were in a committed relationship (*n* = 35, 85.4%), with a university degree (*n* = 29, 70.7%), and in paid employment (*n* = 31, 75.6%). Almost one-third of parents were either themselves autistic (*n* = 6, 14.6%) or questioning if they were (*n* = 6, 14.6%).

**Table 1:** Demographics of participants by group

		Intervention (n = 20)	Care as Usual (n = 21)
Variables		*M(SD)*	*M(SD)*
Child’s age (years)		6.35(2.39)	6.48(2.60)
Parent’s age (years)		40.55(6.43)	38.90(4.77)
		*%(n)*	*%(n)*
Child Sex	Male	65.0(13)	81.0(17)
	Female	35.0(7)	19.0(4)
Parent Sex	Male	15.0(3)	9.5(2)
	Female	85.0(17)	90.5(19)
Parent Country of Origin	Australia	70.0(14)	81.0(17)
	India	–	4.8(1)
	Iraq	–	4.8(1)
	Malaysia	5.0(1)	4.8(1)
	South Africa	–	4.8(1)
	China	5.0(1)	–
	Colombia	5.0(1)	–
	United Kingdom	15.0(3)	–
Parent’s relationship status	Single	5.0(1)	14.3(3)
	In a committed relationship	90.0(18)	81.0(17)
	Separated/divorced	5.0(1)	4.8(1)
Parent’s highest education	High School	–	9.5(2)
	Vocational Certificate	15.0(3)	33.3(7)
	University degree	85.0(17)	57.1(12)
Parent’s employment	Full time (~ 38 h/week)	20.0(4)	28.6(6)
	Part time/Casual	65.0(13)	52.4(11)
	Not employed	15.0(3)	19.0(4)
Parent’s autism diagnosis	Formally diagnosed	10.0(2)	9.5(2)
	Self-identified	5.0(1)	4.8(1)
	Not sure – questioning	20.0(4)	9.5(2)
	Not autistic	65.0(13)	76.2(16)
If autistic, did they know they were as a child?	No	100.0(7)	100.0(5)
	Yes	–	–

### Program Use and Attrition

The AutInsight sessions were held online via Zoom from September 2023 to July 2024. Parents who were not able to attend a particular session due to sickness or unforeseen circumstances were encouraged to join another group for that session or arrange a 1-to-1 catch-up with the facilitator. Overall, 14.6% of families (*n* = 6) were lost to follow up after T1 (AutInsight = 20.0%, 4; CAU = 9.5%, 2). Of parents who were allocated to the AutInsight group, 85.0% (*n* = 17) received and completed 4–5 sessions, 10.0% (*n* = 2) received three sessions or fewer and were not able to arrange for catch-up sessions, and 5.0% (*n* = 1) did not receive the AutInsight program as they could no longer commit the time for sessions. Of parents who received the program, one declined to complete T2 assessment due to burnout, while three were not contactable. Of parents who were allocated to the CAU group (*n* = 21), one parent withdrew prior to T2, and another was uncontactable. Another 14.6% of families (*n* = 6) were lost to follow up at T3 (AutInsight = 10%, 2; CAU = 20%, 4). Of parents who were allocated to the AutInsight group, two were not contactable. Of the parents who were allocated to the CAU group, two withdrew before T3 and another two were not contactable. Overall, this amounted to a total attrition of 31.7% overall (AutInsight = 35%, 7; CAU = 28.6%, 6), with a total of 28 parents completing all aspects of the study (AutInsight = 13; CAU = 15). No adverse effects, concerns or unintended consequences were identified.

**Intervention acceptability, fidelity and feasibility.** The feedback survey was only provided to participants who were allocated to the AutInsight condition (*n* = 20). Parents who completed the survey (*n* = 16) said that they were very likely to recommend this program to another parent (*M* = 8.75, *SD* = 1.39) on a scale of 1–10, and that the program was very neurodiversity-affirming (*M* = 9.44, *SD* = 1.03). Most parents (81.3%, *n* = 13) reported that they felt they now had greater insight into their child, with the rest (18.8%, *n* = 3) reporting that they felt their insight had stayed the same. Most parents reported that they felt they were now more accepting of their child (73.3%, *n* = 11), with the rest reporting that they felt their acceptance had stayed the same (26.7%,* n* = 4). Most parents, however, reported that their self-care had mostly stayed the same (68.8%, *n* = 11), with some reporting that they had increased their self-care (25.0%, *n* = 4), and one parent reporting they have less self-care (6.30%,* n* = 1).

Parents were also asked at the end of the feedback survey about anything they would change about the program. Parents provided a range of logistical feedback around session duration (e.g., shorter, or longer sessions), organising groups by child’s age, as well as receiving some prompts in between sessions to reflect on the week’s material. Qualitative feedback suggested that parents who found the AutInsight program beneficial enjoyed hearing different perspectives from other parents (autistic or non-autistic) and found that the program supported them to “refocus on the little things” as well as learn about their own parenting triggers and beliefs. Parents who were already familiar with some of the content reported developing a deeper and renewed understanding of autism and acceptance. For instance, one parent said that the program helped them to reframe their child’s behaviours, “understanding that he really just wants acceptance and love, and his actions aren’t directed to me”. Other feedback included suggestions that grouping parents based on their child’s support needs and/or demographics (e.g., child age) could be helpful. Qualitative feedback from parents will be further discussed in a separate paper.

Fidelity was examined for each session by whether the session learning objectives were met and was determined by having an independent researcher rate whether each learning objective of each session was met, partially met, or not met. Overall, 100% of content was covered, with 100% agreement between the facilitator and a researcher not involved in program delivery.

Feasibility was examined by the number of families who assessed the study website, recruitment and retention rates. During the recruitment period from September 2023–February 2024, a total of 793 views (608 unique users) were recorded on the study website. In terms of recruitment, a total of 61 parents contacted the first author for information. Out of those who had contacted the first author, 51 parents were assessed for eligibility. Out of those who were assessed for eligibility, 41 parents were randomised into the study. Out of those who were allocated to the AutInsight group (*n* = 20), 85% (*n* = 17) attended all 5 sessions (or attended a catch-up session if they had missed a session). 10% (*n* = 2) attended 3 or less sessions, and 5% (*n* = 1) did not attend any sessions and were not contactable. Three parents attended with their partners.

### Intervention Effects

Means, standard deviations, and effect sizes (Cohen’s *d*) are presented in Table 2. Results of MMRM analyses assessing rate of change across all assessment timepoints (T1–T3) are presented in Table 3. Visual depictions of rate of change over time based on regression parameters are presented in Supplementary Materials.

**Table 2:** Means and standard deviations for outcome variables by treatment condition and effect sizes

		Intervention			Care as usual			Effect size	
		T1	T2	T3	T1	T2	T3	T1-T2	T1-T3
Measure	α	M (SD)	M (SD)	M (SD)	M (SD)	M (SD)	M (SD)	d [95% CI]	d [95% CI]
EAS^a^									
Sensitivity	–	25.30(3.05)	24.53(2.36)	26.13(2.45)	26.76(2.45)	26.53(3.43)	26.93(2.91)	− 0.19[− 0.87, 0.49]	0.23[− 0.47, 0.93]
Structuring	–	26.05(2.56)	24.93(2.79)	26.40(2.03)	26.71 (3.05)	26.59(3.43)	26.67(2.58)	− 0.34[− 1.02, 0.34]	0.14[− 0.56, 0.83]
Non-Intrusive	–	25.20(3.46)	25.00(2.90)	26.40(2.50)	25.95 (3.65)	26.24(4.93)	26.93(2.15)	− 0.13[− 0.81, 0.55]	0.06[− 0.64, 0.76]
Non-hostility	–	28.30(1.84)	28.47(1.06)	28.47(1.06)	28.48 (0.92)	28.76(0.97)	28.87(0.35)	− 0.08[− 0.76, 0.59]	0.12[− 0.58, 0.81]
Child responsiveness	–	26.00(2.68)	24.80(2.65)	25.27(2.94)	26.04 (3.60)	26.71(2.85)	25.93(3.94)	− 0.57[− 1.26, 0.12]	− 0.19[− 0.89, 0.51]
Child Involvement	–	24.45(3.17)	23.20(3.67)	23.33(3.64)	25.48 (3.36)	25.24(4.07)	24.00(4.41)	− 0.30[− 0.98, 0.38]	0.11[− 0.59, 0.80]
EA-SR									
Mutual Attune^b^	.77	27.75(5.97)	29.81(4.79)	32.50(5.61)	29.86 (6.29)	28.95(5.71)	29.33(6.69)	0.47[− 0.19, 1.13]	0.84[0.10, 1.57]
Child Involve^c^	.88	36.10(7.11)	36.75(5.76)	37.86(6.05)	38.50 (4.39)	38.05(3.73)	37.27(6.47)	0.18 [− 0.47, 0.84]	0.50[− 0.22, 1.21]
Intrusiveness^d^	.64	18.75(2.75)	19.19(3.08)	18.79(2.72)	18.81 (4.84)	19.16(2.87)	18.73(4.03)	− 0.02[−0.67, 0.63]	− 0.03[−0.74, 0.68]
Hostility^e^	.94	20.30(6.71)	18.63(5.94)	17.43(6.69)	20.05 (6.75)	20.05(6.75)	20.87(7.00)	0.24[− 0.41, 0.90]	0.53[− 0.19, 1.25]
PAUACS^f^	.83	180.93(13.65)	184.69(16.39)	187.07(10.76)	186.30(14.63)	188.32(11.49)	188.60(12.64)	0.12[− 0.53, 0.77]	0.26[− 0.45, 0.97]
Understanding	.74	76.81(4.60)	77.06(5.04)	76.79(4.98)	78.10(4.78)	78.05(5.13)	77.80(5.94)	0.06[− 0.59, 0.71]	0.06[− 0.65, 0.76]
Innate	.75	31.75(6.32)	34.44(5.56)	34.43(4.33)	34.52(6.53)	35.21(5.05)	35.33(4.32)	0.30[− 0.35, 0.96]	0.28[− 0.43, 0.99]
Acceptance	.77	30.05(4.51)	30.50 (4.15)	31.64(3.48)	31.16(4.16)	31.32(3.54)	31.60(3.70)	0.07[− 0.58, 0.72]	0.26[− 0.45, 0.97]
BMPS^g^	.85	27.50(6.24)	27.56(4.63)	29.64(4.86)	28.24(7.38)	27.95(6.97)	28.40(6.99)	0.05[− 0.60, 0.70]	0.28[− 0.43, 0.99]
comPACT^c^	.89	57.05(21.13)	60.56 (23.59)	57.64(21.19)	55.00(22.32)	58.26(24.77)	54.87(23.92)	− 0.01[− 0.66, 0.64]	− 0.03[− 0.74, 0.68]
Open to Experience	.85	27.40(11.57)	29.00 (9.54)	27.50(9.25)	29.00(12.74)	29.74(13.21)	28.40(12.39)	− 0.07[− 0.72, 0.58]	− 0.06[− 0.76, 0.65]
Behavioral Awareness	.83	15.25(5.78)	16.69 (6.76)	16.14(6.79)	14.81(7.47)	16.79(8.26)	16.53(7.53)	0.08[− 0.57, 0.73]	0.12[− 0.59, 0.83]
Valued Action	.82	14.40(8.06)	14.88 (10.76)	14.00(10.50)	11.19(6.53)	11.74(8.29)	9.93(7.17)	0.01[− 0.64, 0.66]	− 0.11[− 0.82, 0.59]
DASS-42^ h^									
Depression	.95	11.20(10.90)	10.88 (10.53)	12.00(12.75)	10.48(11.04)	9.68(10.29)	11.07(12.87)	− 0.04[− 0.69, 0.61]	− 0.02[− 0.73, 0.69]
Anxiety	.91	8.20(8.78)	6.50 (9.45)	7.57(9.19)	9.81(12.18)	9.47(10.97)	8.13(11.99)	0.12[− 0.53, 0.77]	− 0.10[− 0.80, 0.61]
Stress	.92	17.50(11.91)	16.75(9.88)	17.57(11.10)	18.57(9.45)	18.21(12.59)	17.73(12.83)	0.04[− 0.61, 0.69]	− 0.08[− 0.79, 0.63]
SDQ^i^									
Emotional Problems	.72	3.90(2.47)	3.69(2.96)	2.86(2.35)	4.86(2.65)	4.26(2.70)	3.60(2.16)	− 0.14[− 0.80, 0.51]	− 0.08[− 0.79, 0.63]
Conduct Problems	.60	3.70(2.03)	3.19(2.10)	2.50(1.65)	4.00(2.37)	4.53(2.19)	4.20(2.14)	0.46[− 0.20, 1.12]	0.62[− 0.11, 1.34]
Hyperactivity	.74	6.95(2.24)	7.06(1.88)	5.86(2.25)	7.29(2.33)	7.24(2.48)	6.87(1.81)	− 0.07[− 0.72, 0.58]	0.29[− 0.42, 1.00]
Peer Probroms	.59	3.10(2.17)	3.63(2.00)	2.79(1.93)	4.52(2.20)	4.22(2.27)	4.07(1.83)	− 0.37[− 1.03, 0.28]	− 0.06[− 0.77, 0.64]
Prosocial	.81	4.50(2.63)	4.31(2.73)	5.21(3.45)	6.57(2.50)	5.93(2.31)	6.00(2.00)	0.17[− 0.48, 0.82]	0.48[− 0.23, 1.21]
BFRS-R^j^	.88	14.05(5.45)	14.44(5.90)	12.50(4.47)	15.33(7.01)	15.06(6.53)	12.87(5.64)	− 0.10[− 0.76, 0.56]	− 0.14[− 0.85, 0.57]
Flex with Objects	.83	10.00(3.71)	9.50(4.03)	8.93(2.84)	10.14(3.95)	9.72(3.91)	8.87(3.48)	0.02[− 0.64, 0.68]	− 0.05[− 0.76, 0.66]
Flex with Environments	.80	2.85(1.69)	3.38(1.63)	2.43(1.60)	3.81(2.84)	4.06(2.15)	2.73(1.75)	− 0.11[− 0.77, 0.54]	− 0.27[− 0.98, 0.44]
Flex with Persons	.61	1.20(1.24)	1.56(1.21)	1.14(1.10)	1.38(1.16)	1.28(1.23)	1.27(1.03)	− 0.38[− 1.04, 0.28]	− 0.05[− 0.75, 0.66]
AFEQ^k^	.92	134.33(22.65)	128.50(23.11)	124.29(25.29)	131.14(17.38)	131.50(16.34)	128.87(19.70)	0.30[− 0.36, 0.96]	0.38[− 0.34, 1.09]
Family Life	.81	26.40(5.86)	25.19(7.32)	24.00(6.97)	26.52(5.72)	28.17(5.96)	25.87(5.14)	0.48[− 0.18, 1.15]	0.29[− 0.42, 1.00]
Experience of Parent	.89	36.43(6.57)	35.25(6.51)	32.50(7.22)	34.90(7.91)	33.06(7.63)	33.73(8.84)	− 0.09[− 0.75, 0.57]	0.37[− 0.35, 1.08]
Child Symp	.69	34.40(5.44)	32.06(5.69)	31.64(6.33)	33.24(4.57)	33.44(3.81)	32.80(3.95)	0.50[− 0.17, 1.16]	0.45[− 0.27, 1.17]
Child Dev	.82	37.10(8.50)	36.00(7.78)	36.14(8.24)	36.48(6.90)	36.83(6.46)	36.47(7.99)	0.18[− 0.47, 0.84]	0.12[− 0.59, 0.83]
QoLA- A^l^	.96	88.65(21.18)	89.13(19.68)	88.00(26.00)	93.76(25.28)	91.67(25.40)	94.13(25.57)	0.11[− 0.55, 0.77]	− 0.04[− 0.75, 0.67]
QoLA- B^m^	.92	67.00(17.14)	70.31(15.09)	71.93(17.11)	65.19(17.88)	64.72(16.05)	71.07(14.66)	0.21[− 0.45, 0.87]	− 0.05[− 0.76, 0.66]
Overall QoLA^n^		5.55(2.35)	5.63(2.66)	5.50(2.95)	5.95(2.13)	5.67(2.54)	5.67(2.66)	0.16[− 0.50, 0.82]	0.10[− 0.61, 0.81]

α = Cronbach’s α, EAS = Emotional Availability Scales (Observation), EA-SR = Emotional Availability (Self-report), PAUACS = Parental acceptance and understanding of autistic children scale, BMPS = Bangor Mindful Parenting Scale, compACT = Comprehensive Assessment of Acceptance and Commitment Therapy Processes, DASS-42 = Depression Anxiety and Stress Scales-42, SDQ = Strengths and Difficulties Questionnaire, BFRS-R = Behavior Flexibility Rating Scale-revised, AFEQ = Autism Family and Experience Questionnaire, QoLA = Quality of Life Autism

^a^EAS has 7 scales, each with a range of 7–29. Higher scores represent higher levels of observed emotional availability. As this is an observational scale, no Cronbach’s alpha was calculated for these scales. ^b^Mutual Attunement (EA-SR) has a range of 10–50, higher scores represent higher levels of self-reported mutual attunement. ^c^Child Involvement (EA-SR) has a range of 9–45, higher scores represent higher levels of self-reported child involvement. ^d^Intrusiveness (EA-SR) has a range of 6–30, higher scores represent higher levels of self-reported intrusiveness. ^e^Hostility (EA-SR) has a range of 6–30, higher scores represent higher levels of self-reported hostility. ^f^PAUACS has a range of 30–210, higher scores represent higher levels of acceptance and understanding. ^g^BMPS has a range of 0–45, higher scores represent higher levels of parental mindfulness. compACT has a range of 0–138, higher scores represent greater psychological flexibility^. h^DASS-42 scores were derived by multiplying Depression Anxiety Stress Scales (DASS-21) scores by 2 in order to achieve comparability. Range = 0–42. Higher scores represent higher levels of symptoms. Moderate, severe, or extremely severe symptoms represent clinically significant symptoms. ^i^SDQ has 5 scales with 5 items each. Each scale ranges from 0 to 10, higher scores represent higher levels of behaviors. ^j^BFRS-R has a range of 0–32, higher scores represent greater behavioral inflexibility. ^k^AFEQ has a range of 48–240, higher scores represent more challenging experience. ^l^QoLA-A has a range of 28–140, higher scores represent greater perceived QoL. ^m^QoLA-B has a range of 20–100, higher scores represent fewer problems for parents relating to autism-related behaviors. ^n^Overall QoLA is a single 10-point Likert scale with a range of 0–10, higher scores represent greater overall quality of life

**Table 3:** Intervention effects for outcome variables

Model	1				2				3		4			
	Estimate of fixed effects: time				Estimate of fixed effects: time X condition interaction				Estimate of fixed effects: time (separate by condition)				Comparison	
									Intervention		Care as Usual			
Measure	B	*F*	*df*	*p*	B	*F*	*df*	*p*	B	*p*	B	*p*	*t*	*p*
Parental sensitivity [H1]														
EAS (Sensitivity)	0.20	0.45	71.26	.51	0.31	0.26	71.45	.61	0.37	.36	0.06	.89	− 0.51	0.61
EAS (Structuring)	0.04	0.02	73.10	.89	0.22	0.14	72.58	.71	0.16	.67	− 0.06	.89	− 0.38	0.70
EAS (Non-Intrusiveness)	0.54	2.47	72.42	.12	0.13	0.04	72.32	.85	0.62	.14	0.47	.39	− 0.21	0.83
EAS (Non-Hostility)	0.28	1.40	31.45	.25	0.08	0.10	72.75	.76	0.28	.21	0.19	.12	− 0.34	0.73
EAS (Child responsiveness)	− 0.22	0.58	71.41	.45	− 0.42	0.37	34.05	.55	− 0.34	.30	− 0.07	.88	0.47	0.64
EAS (Child involvement)	− 0.57	3.33	70.49	.07	0.29	0.18	33.21	.68	− 0.39	.34	− 0.73	.13	− 0.54	0.59
EA-SR (Mutual Attunement)	0.79	3.36	66.31	.071	2.31	7.95	66.54	.006^**^	1.99	.001^**^	− 0.32	.593	− 2.83	.007^**^
EA-SR (Child Involvement)	0.39	1.36	67.02	.248	1.82	8.43	66.54	.005^**^	1.36	.008^**^	− 0.48	.244	− 2.92	.006^**^
EA-SR (Intrusiveness)	− 0.02	0.004	69.30	.949	− 0.05	0.01	34.88	.939	− 0.09	.770	0.03	.957	0.19	.851
EA-SR (Hostility)	− 0.42	2.01	65.34	.161	− 1.27	3.62	34.98	.065	− 1.14	.049^*^	0.18	.664	1.96	.058
Parental Acceptance and Understanding [H2]														
PAUACS	1.55	3.73	66.26	.058	2.27	2.02	31.98	.165	2.71	.053	0.51	.615	− 1.36	.183
Understanding	− 0.05	0.02	67.58	.881	0.09	0.02	30.57	.898	− 0.07	.905	− 0.10	.801	− 0.05	.964
Innate	0.74	4.65	65.81	.035^*^	1.04	1.85	34.47	.183	1.33	.024^*^	0.27	.623	− 1.37	.178
Acceptance	0.17	0.41	64.89	.525	0.51	0.99	64.98	.323	0.42	.270	− 0.07	.839	− 0.96	.344
Parental Psychological Flexibility [H3]														
compACT	0.95	0.81	65.85	.371	− 1.88	0.81	65.83	.372	− 0.03	.982	1.84	.231	0.90	.372
Openness to Experience	0.18	0.08	65.72	.776	− 0.79	0.39	65.76	.532	− 0.22	.800	0.56	.538	0.63	.534
Behavioural Awareness	0.97	6.24	67.44	.015^*^	− 0.82	1.14	67.40	.290	0.54	.266	1.35	.027	1.08	.288
Valued Action	− 0.23	0.19	67.07	.663	− 0.18	0.03	67.20	.861	− 0.32	.659	− 0.17	.819	0.14	.890
Parental Mindful Parenting [H4]														
BMPS	0.37	0.97	65.74	.328	0.85	1.13	30.28	.295	0.86	.158	− 0.03	.959	− 1.12	.271
Parental Mental Health [H5]														
DASS-42														
Depression	0.69	2.67	65.02	.107	0.19	0.05	65.05	.828	0.78	.264	0.61	.246	− 0.20	.841
Anxiety	− 0.40	0.46	67.81	.502	− 0.18	0.02	31.51	.902	− 0.37	.656	− 0.07	.952	0.20	.841
Stress	0.04	0.004	66.00	.951	0.20	0.03	66.03	.863	0.13	.889	− 0.06	.939	− 0.16	.875
Autism family experience [H6]														
AFEQ	− 2.10	4.43	65.18	.039^*^	− 4.90	5.31	32.92	.028^*^	− 4.59	.002^**^	0.24	.888	2.31	.027^*^
Family Life	− 0.31	1.06	65.02	.307	− 1.45	6.00	32.67	.020^*^	− 1.03	.036^*^	0.38	.351	2.36	.024^*^
Parent Experience	− 0.85	3.93	65.02	.052	− 1.36	2.60	65.05	.112	− 1.56	.010^*^	− 0.19	.755	1.63	.111
Child symptoms	− 0.62	3.71	67.03	.058	− 1.20	2.59	33.22	.117	− 1.19	.008^**^	− 0.09	.889	1.52	.136
Child Development	− 0.38	1.13	65.38	.291	− 0.92	1.77	65.32	.189	− 0.87	.078	0.06	.899	1.35	.184
Quality of Life [H7]														
Quality of Life Autism- A	− 0.45	0.25	64.57	.621	1.80	0.99	64.49	.324	0.43	.780	− 1.34	.207	− 0.96	.341
Quality of Life Autism- B	2.42	4.35	67.91	.041^*^	1.32	0.33	68.05	.570	3.13	.063	1.81	.279	− 0.57	.571
Overall Quality of Life Autism	− 0.15	0.89	66.71	.349	0.34	1.26	66.53	.265	0.03	.904	− 0.32	.098	− 1.13	.268
Parent-reported Child Adjustment [H8]														
SDQ (Emotional Problems)	− 0.52	6.71	70.57	.012^*^	0.15	0.12	39.64	.729	− 0.43	.127	− 0.60	.085	− 0.41	.685
SDQ (Conduct Problems)	− 0.17	1.45	67.61	.233	− 0.58	4.64	67.39	.035^*^	− 0.49	.030^*^	0.10	.522	2.21	.033^*^
SDQ (Hyperactivity)	− 0.36	5.19	67.38	.026^*^	− 0.24	0.56	67.33	.458	− 0.49	.066	− 0.27	.171	0.71	.484
SDQ (Peer problems)	− 0.18	1.21	66.46	.276	− 0.19	0.36	67.70	.550	− 0.29	.185	− 0.10	.683	0.61	.545
SDQ (Prosocial)	− 0.04	0.06	66.38	.802	0.94	8.05	34.60	.008^**^	0.46	.098	− 0.48	.034^*^	− 2.81	.008^**^
Parent-reported Child Behavioural Flexibility [H9]														
BFRS-R	− 0.99	5.60	66.46	.021^*^	0.39	0.21	66.57	.646	− 0.78	.246	− 1.19	.030^*^	− 0.50	.623
Flexibility with Objects	− 0.66	6.10	66.69	.016^*^	− 0.01	0.001	66.78	.992	− 0.66	.115	− 0.67	.070	− 0.02	.985
Flexibility with Environments	− 0.28	2.79	66.98	.099	0.38	1.27	66.48	.263	− 0.12	.610	− 0.47	.044^*^	− 1.04	.304
Flexibility with Persons	− 0.05	0.23	68.19	.637	0.02	0.006	68.34	.936	− 0.04	.818	− 0.05	.672	− 0.09	.926

PAUACS = Parental acceptance and understanding of autistic children scale, BMPS = Bangor Mindful Parenting Scale, compACT = Comprehensive Assessment of Acceptance and Commitment Therapy Processes, DASS-42 = Depression Anxiety and Stress Scales-42, SDQ = Strengths and Difficulties Questionnaire, BFRS-R = Behavior Flexibility Rating Scale-revised, AFEQ = Autism Family and Experience Questionnaire, QoLA = Quality of Life Autism

^*^p < .05, ** *p* < .01

### Intervention Effects: Primary Outcomes

**Emotional Availability (EAS) [H1].** Time did not predict change in observed sensitivity, structuring, non-intrusiveness, non-hostility, child responsiveness or child involvement, and a lack of time-by-group interaction indicated no statistically significant condition-level intervention effect (Table 3).

**Emotional Availability (EA-SR) [H1].** Time did not predict change in Mutual Attunement or Child Involvement (Table 3). A statistically significant time-by-condition interaction indicated medium to large intervention effects (Mutual attunement; *d* = 0.84 at T3, Child Involvement; *d* = 0.50 at T3; Table 2). Follow-up contrasts showed greater rate of change (increase) for both subscales for the AutInsight group, which was sustained at follow-up, as compared to CAU, which showed no change (Table 3). Time did not predict change in self-reported Intrusiveness or Hostility, and a lack of time-by-group interaction indicated no statistically significant condition-level intervention effect.

### Intervention Effects: Secondary Outcomes

**Parental Acceptance and Understanding (PAUACS)[H2].** Time did not predict change in parental acceptance and understanding (PAUACS total score) over time and lack of time-by-condition interaction indicated no statistically significant intervention effect. For PAUACS subscales, time predicted change (increase) in the subscale Innate over time. The lack of time-by-condition interaction again indicated no statistically significant condition-level intervention effect (Innate; small effect *d* = 0.28 at T3; Table 2)*.* While follow up contrasts found no statistically significant difference between conditions on rate of change, the AutInsight group had a statistically significant improvement over time whereas CAU showed no change. Time did not predict change in subscales Understanding or Acceptance, and lack of time-by-group interactions indicated no statistically significant condition-level intervention effects.

**Parental Psychological Flexibility (comPACT) [H3].** Time predicted change (increase) in present moment awareness (comPACT; Behavioral Awareness*;* Table 3). The lack of a time-by-condition interaction indicated no statistically significant condition-level intervention effect (Behavioural Awareness; small effect *d* = 0.12 at T3; Table 2). Time did not predict change in Openness to Experience and Valued Action, and a lack of a time-by-condition interaction indicated no statistically significant condition-level intervention effect.

**Parental Mindful Parenting (BMPS) [H4].** Time did not predict change in mindful parenting (BMPS), and a lack of time-by-group interaction indicated no statistically significant condition-level intervention effect.

**Parental Mental Health (DASS-42) [H5].** Time did not predict changes in depression, anxiety, or stress scores, and a lack of time-by-group interactions indicated no statistically significant condition-level intervention effects (Table 3).

***Depression.*** Overall, 14.3% (1/7) of parents in the AutInsight group who were in the clinical range for depression at T1 moved into the non-clinical range by T2, χ^2^ (1, *n* = 16) = 8.91, *p* = 0.003, compared to 12.5% (1/8) of those in the CAU group, χ^2^ (1, *n* = 19) = 15.24, *p* < 0.001. At T3, 0.0% (0/6) of parents in the AutInsight group who were in the clinical range at T1 had moved into the non-clinical range, χ^2^ (1, *n* = 14) = 14.00, *p* < 0.001, compared to 16.7% (1/6) of those in the CAU group, χ^2^ (1, *n* = 15) = 11.25, *p* = 0.001. Chi-squared tests for independence indicated a greater proportion of participants in the AutInsight group showed clinical improvement in depression scores from T1-T2 compared to CAU, however, this was not sustained to T3.

***Anxiety.*** Overall, 37.5% (3/8) of parents in the AutInsight group who were in the clinical range for anxiety at T1 moved into the non-clinical range by T2, χ^2^ (1, *n* = 16) = 7.27, *p* = 0.007, compared to 14.3% (1/7) of those in the CAU group, χ^2^ (1, *n* = 19) = 6.54, *p* = 0.011. At T3, 33.3% (2/6) of parents in the AutInsight group who were in the clinical range at T1 had moved into the non-clinical range, χ^2^ (1, *n* = 14) = 7.47, p = 0.006, compared to a non-significant change of 50.0% (2/4) of those in the CAU group, χ^2^ (1, *n* = 15) = 0.68, *p* = 0.41. Chi-squared tests for independence indicated a greater proportion of participants in the AutInsight group showed clinical improvement in anxiety scores from T1-T3.

***Stress.*** Overall, 14.3% (1/7) of parents in the AutInsight group who were in the clinical range for stress at T1 moved into the non-clinical range by T2, χ^2^ (1, *n* = 16) = 6.35, *p* = 0.012, compared to 0.0% (0/9) of those in the CAU group, χ^2^ (1, *n* = 19) = 15.39, *p* < 0.001. At T3, 0.0% (0/6) of parents in the AutInsight group who were in the clinical range at T1 had moved into the non-clinical range, χ^2^ (1, *n* = 14) = 10.50, p = 0.001, compared to 16.7% (1/6) of those in the CAU group, χ^2^ (1, *n* = 15) = 7.82, *p* = 0.005. Chi-squared tests for independence indicated a greater proportion of participants in the AutInsight group showed clinical improvement in stress scores from T1-T2, however, this was not sustained to T3.

**Autism Family Experience (AFEQ) [H6].** Time predicted change (improvement) in overall family experience (AFEQ total score; Table 3), and a statistically significant time-by-group interaction indicated a small condition-level intervention effect (AFEQ total; small effect *d* = 0.38 at T3; Table 2). Follow-up contrasts showed greater rate of change (decrease) for the AutInsight group as compared to CAU, which showed no change (Table 3). For AFEQ subscales, time did not predict change in the subscale Family Life, however, a significant time-by-group interaction indicated a small condition-level intervention effect (Family Life; small effect *d* = 0.29 at T3; Table 2). Follow-up contrasts showed greater rate of change (decrease) for the AutInsight group as compared to CAU, which showed no change (Table 3). Time did not predict change in the subscales Parent Experience or Child Symptoms, and a lack of a time-by-condition interactions indicated no significant condition-level intervention effects. While follow-up contrasts were also not statistically significant, the AutInsight group had a significant rate of change (decreases) for both subscales as compared to CAU, which showed no change (Table 3). Time did not predict change in parent-reported Child Development, and a lack of time-by-group interaction indicated no statistically significant condition-level intervention effect (Table 3).

**Quality of Life in Autism (QoLA)[H7].** Time predicted change (increase) in the impact of autism symptoms**.** The lack of time-by-group interaction indicated no statistically significant condition-level intervention effect (Table 3). Time did not predict change in overall quality of life (QoLA-A; Overall QoL) and a lack of time-by-group interaction indicated no statistically significant condition-level intervention effect (Table 3).

**Parent-reported Child Adjustment (SDQ)[H8].** Time did not predict change in conduct problems. A statistically significant time-by-condition interaction indicated medium intervention effects (Conduct Problems [SDQ]; *d* = 0.62 at T3; Table 2). Follow-up contrasts showed greater rate of change (decrease) for the AutInsight group as compared to CAU, which showed no change (Table 3). Overall, 25.0% (2/8) of children in the AutInsight group who were in the clinical range for conduct problems at T1 moved into the non-clinical range by T2, χ^2^ (1, *n* = 16) = 6.35, *p* = 0.01, compared to 7.7% (1/13) of those in the CAU group, χ^2^ (1, *n* = 18) = 13.85, *p* < 0.001. At T3, 42.9% (3/7) of children in the AutInsight group who were in the clinical range at T1 had moved into the non-clinical range, χ^2^ (1, *n* = 14) = 5.60, *p* = 0.02, compared to 0.0% (0/10) of those in the CAU group, χ^2^ (1, *n* = 15) = 15.00, *p* < 0.001. Chi-squared tests for independence indicated a greater proportion of participants in the AutInsight group reported clinical improvement in conduct problems in their children from T1-T3.

Time did not predict change in prosocial behaviour, however, a statistically significant time-by-condition interaction indicated medium intervention effects (Prosocial Behaviour [SDQ]; *d* = 0.48 at T3; Table 2). Follow-up contrasts showed greater rate of change (decrease) for the CAU group as compared to the AutInsight group, which showed no change (Table 3). Similarly, 18.2% (2/11) of children in the CAU group who were in the normal range for prosocial behavior at T1 moved to the clinical range by T2, χ^2^ (1, *n* = 19) = 12.44, *p* < 0.001, as compared to 33.3% (1/3) in the AutInsight group, χ^2^ (1, *n* = 16) = 2.16, *p* = 0.14. At T3, 40.0% (4/10) of children in the CAU group who were in the normal range at T1 had moved to the clinical range, χ^2^ (1, *n* = 15) = 5.00, *p* = 0.03, compared to 33.3% (1/3) in the AutInsight group, χ^2^ (1, N = 14) = 0.88, *p* = 0.35. Chi-squared tests for independence indicated a greater proportion of participants in the CAU group reported clinical reduction in prosocial behavior in their children from T1-T3, as compared to the AutInsight group which showed no change.

Time predicted change in parent-reported child emotional problems and hyperactivity (Table 3). A lack of time-by-group interaction indicated no statistically significant condition-level intervention effect (Emotional Problems [SDQ]; no effect at T3, Hyperactivity [SDQ]; small effect *d* = 0.29 at T3; Table 2). Similarly, 27.3% (3/11) of children in the CAU group who were in the clinical range for emotional problems at T1 moved into the non-clinical range at T2, χ^2^ (1, *n* = 19) = 6.74, *p* = 0.01, while there was no change in the AutInsight group (33.3% (1/3); χ^2^ (1, *n* = 16) = 2.16, *p* = 0.14). At T3, 25.0% (2/8) of children in the CAU group who were in the clinical range at T1 had moved into the non-clinical range, χ^2^ (1, *n* = 15) = 5.53, *p* = 0.02, however, there were no change in the AutInsight group (66.7% (2/3); χ^2^ (1, *n* = 14) = 1.13, *p* = 0.29). Chi-squared tests for independence indicated no clinical improvement in emotional problems of their children of parents in the AutInsight group from T1-T3.

Overall, 20.0% (1/5) of children in the AutInsight group who were in the clinical range for hyperactivity problems at T1 moved into the non-clinical range by T2, χ^2^ (1, *n* = 16) = 3.88, *p* = 0.049, compared to 27.3% (3/11) of those in the CAU group, χ^2^ (1, *n* = 19) = 6.74, *p* = 0.01. At T3, there were no significant movement of children who were in the clinical range at T1 that moved into the non-clinical range in either the AutInsight group (75.0% (3/4); χ^2^ (1, *n* = 14) = 0.04, *p* = 0.84) or the CAU group (55.6% (5/9); χ^2^ (1, *n* = 15) = 3.64, *p* = 0.06). Chi-squared tests for independence indicated a greater proportion of participants in the AutInsight group reported clinical improvement in hyperactivity problems in their children from T1-T2, however this was not sustained to T3.

Time did not predict change in peer problems [SDQ], and a lack of time-by-group interaction indicated no statistically significant condition-level intervention effect (Table 3). Overall, 25.0% (2/8) of children in the AutInsight group who were in the clinical range for peer problems at T1 moved into the non-clinical range by T2, χ^2^ (1, *n* = 16) = 6.35, *p* = 0.01, compared to a non-significant change of 23.1% (3/13) of those in the CAU group, χ^2^ (1, *n* = 19) = 3.35, *p* = 0.07. However, this did not sustain to T3 for children in the AutInsight group, (57.1%4/7; χ^2^ (1, *n* = 14) = 0.31, *p* = 0.58), as well as those in the CAU group, (20.0% (2/10); χ^2^ (1, *n* = 15) = 2.40, *p* = 0.12).

**Parent-reported Child Behaviour Flexibility (BFRS-R) [H9].** Time predicted change in parent-reported child behaviour flexibility (Table 3). A lack of time-by-group interaction indicated no statistically significant condition-level intervention effect (BFRS-R; small effect *d* = − 0.14 at T3; Table 2). Time predicted change (increase) in child Flexibility with Objects (Table 3), however, a lack of time-by-group interaction indicated no statistically significant condition-level intervention effect (Table 2). Time did not predict change in child Flexibility with Environments or Flexibility with Persons, and a lack of time-by-group interaction indicated no statistically significant condition-level intervention effect (Table 3).

## Discussion

This study outlines the development and initial evaluation of AutInsight—an online innovative consumer-informed parent support program that is grounded in the perspectives of autistic adults (Lee et al., 2023). In terms of primary outcomes, and consistent with hypotheses, parent-reported mutual attunement and child involvement showed improvement following the AutInsight program, with improvements sustained at 3-month follow-up. This is in line with previous research conducted on the development and testing of online parenting programs for parents of autistic children, where parents benefit from a group program focused on developing the parent–child relationship (Poslawsky et al., 2014) as well as parental mental health and wellbeing (Leadbitter et al., 2022). However, contrary to hypotheses, observed parental emotional availability did not change post-program. Self-reported emotional availability represent change in perceptions but is not the same as observed emotional availability (Biringen et al., 2023). In terms of secondary outcomes, overall family experience and parent-reported child conduct problems and prosocial behaviour showed improvement following the program and improvements were sustained at 3-month follow-up. This was consistent with our hypotheses and research that suggests that parent support programs can effect long-term change in parental well-being as well as children’s challenging (Bearss et al., 2018) and prosocial behaviours (Pennefather et al., 2018).

While trends were in the expected directions, this pilot study was not powered to detect small effects. Contrary to expectations, no effects were detected for observed emotional availability, parental acceptance and understanding (i.e., PAUACS scores), mindful parenting, psychological flexibility, psychological adjustment, parental quality of life, or child behavioural flexibility. While some changes in self-reported mutual attunement and child involvement was noted, no change in observed emotional availability was found. This could reflect changes in perceptions, social desirability bias, or that low power resulted in the inability to detect effects in observed emotional availability. In terms of acceptance, a possible reason for the lack of effects could be due to parents being already very accepting of their child at baseline, evident in high baseline scores on the PAUACS, limiting room to shift in acceptance post-program. Further, parents were also mostly well-adjusted at baseline with majority of parents reporting normal levels of depression, anxiety and stress symptoms, which could also explain minimal shifts in psychological adjustment post-program. It is also possible that the reduction in depression and stress symptoms observed post-program could be attributed to the peer support parents experienced while participating in the program, albeit, not sustained at follow-up and not investigated in this pilot trial.

Despite this, results suggest preliminary effectiveness, feasibility and acceptability of the program, and support progression to a larger fully powered trial. Participant feedback suggests that the program was very neurodiversity-affirming and that most parents would recommend the program to another parent of an autistic child. Some parents suggested some logistical tweaks to the program such as arranging groups by child’s age or support needs, which may be more feasible in a larger trial.

### Clinical Implications

This study demonstrates an innovative approach to consumer-informed intervention development where evidence-based approaches are used to develop program components based on what consumers say they want. This study also found that parental sensitivity (EA-SR) can be improved by a parent support program focused on developing parent–child relationship quality. This program could be useful to parents of autistic children who would like to improve the quality of their parent–child relationship in a neurodiversity-affirming group environment. Further, this approach to consumer-informed program development could also be used with other populations, for example families of children with other neurodevelopmental differences such as ADHD.

### Strengths and Limitations

Strengths of the study include that it was neurodiversity-affirming and informed by a reference group of autistic adults as well as grounded in previous qualitative research with autistic adults (Lee et al., 2023). Limitations include that it was a pilot study with a small sample size and therefore underpowered to detect small effects which may be, nevertheless, important to parents and families. Some scales had low internal reliability (e.g. Affect Quality in EA-SR; Vliegen et al., 2009) and had to be excluded from analyses. The sample predominantly comprised highly educated, employed, partnered, Australian-born mothers, and ethnicity was not assessed. Whether results would generalise to samples with different sociodemographic profiles is therefore unknown, and future research should specifically aim to recruit more diverse samples. One clinical psychologist facilitated all groups, which meant that that the therapist was consistent and there was no need to control for therapist effects. However, in a process-based approach where therapeutic style could effect change in itself it is difficult to know if the program or therapist effected change. Finally, most outcome measures including child behavioural outcomes were parent-reported. Given that this program aimed to develop parental acceptance and understanding of their autistic child, it is also difficult to determine if children indeed had improved behavioural outcomes or if parents’ perceptions of their child’s behaviours have changed. Further research is required to determine mechanisms of change. A larger trial is warranted to confirm results.

## Conclusions

Results suggest preliminary feasibility, acceptability and effectiveness of AutInsight in improving parental sensitivity of parents of autistic children. It also demonstrates an innovative consumer-informed approach that could be used to support programs for other populations.

## Supplementary Information

Below is the link to the electronic supplementary material.

Supplementary file1 (DOCX 177 kb)
