# Supplementary material for: AutInsight: A Pilot Randomised Controlled Trial (RCT) of a Consumer-Informed Parent Support Program for Parents of Autistic Children
Source: J Autism Dev Disord. 2025 Feb 27;56(7):2588–605. doi: 10.1007/s10803-025-06764-5 (PMC13346268; doi:10.1007/s10803-025-06764-5)
Supplement: Supplementary file 1 — Supplementary file1 (DOCX 177 kb) [file 10803_2025_6764_MOESM1_ESM.docx]

**Main Topics of Each Session of the Program, Mapped to Themes and Content of Sessions**

| Title | Content |
| --- | --- |
| Session 1: “Your goal is their happiness, not traditional neurotypical success” ^a-j^ | Introduction  The AutInsight program  Psychoeducation and collective understanding of Autism, Social Model of Disability & Neurodiversity  Review and discussion of findings of the qualitative paper that the program was grounded in: *What* did autistic adults say? & *How* it maps with our program? |
| Session 2: Compass & connection^b, c, e^ | **Parenting Values**  Beginning with the end in mind  How do I know what my parenting values are?  **Attachment & Parenting**  What is attachment and why do we need to know about it?  Life experiences can influence parenting.  Parent’s encounters/experience with autism  Meeting your autistic child’s needs from an attachment perspective |
| Session 3: Acceptance & being present^b,c,d,g,i^ | **Acceptance**  Acceptance of what *is* (Radical acceptance)  Acceptance of *internal* experience (Experiential acceptance)  Parental own acceptance as gateway to child acceptance |
| Session 4: Focus on the relationship and what works^b, c, d, g, h^ | Relationship as the foundation    Grounding in needs of child = acceptance + mindfulness + *flexibility*  What works *vs* what should work? |
| Session 5: Review^i^ | Review goals, wins, challenges  Parental burnout & Self-compassion  Goodbyes and new beginnings – what now? |

*Note.* ^1^Themes as identified in Lee, Whittingham, Olson, et al.’s (2023) paper on autistic adults reflections on parenting autistic children. ^a^Diagnosis should be embraced. ^b^The goal is happiness, not neurotypical success. ^c^Unconditional love and acceptance. ^d^Understanding your autistic child is crucial to meeting their needs. ^e^Autistic children have the same basic emotional needs as all children. ^f^Structure, predictability and being explicit is key to comfort and safety at home. ^g^Find a way into your child. ^h^Parents as advocates. ^i^Parental well-being is crucial to child well-being. ^j^Parental neurodivergence can be a bane or a boon.

## **Visual Depictions of Rate of Change over Time Based on Regression Parameters**

**Primary outcomes**

**EAS**

**EA-SR**

**Secondary Outcomes**

**PAUACS**

**BMPS**

**comPACT**

**DASS-42**

**SDQ**

**BFRS-R**

**AFEQ**

**QoLA**
